# Accurate Lipid Quantification of Tissue Homogenates Requires Suitable Sample Concentration, Solvent Composition, and Homogenization Procedure—A Case Study in Murine Liver

**DOI:** 10.3390/metabo11060365

**Published:** 2021-06-08

**Authors:** Marcus Höring, Sabrina Krautbauer, Louisa Hiltl, Verena Babl, Alexander Sigruener, Ralph Burkhardt, Gerhard Liebisch

**Affiliations:** Institute of Clinical Chemistry and Laboratory Medicine, University Hospital of Regensburg, Franz-Josef-Strauß-Allee 11, 93053 Regensburg, Germany; marcus.hoering@ukr.de (M.H.); sabrina.krautbauer@ukr.de (S.K.); louisa.hiltl@stud.uni-regensburg.de (L.H.); verena.babl@klinik.uni-regensburg.de (V.B.); alexander.sigruener@ukr.de (A.S.); ralph.burkhardt@ukr.de (R.B.)

**Keywords:** lipidomics, lipids, extraction, recovery, solvent, quantification, preanalytics, tissue homogenization, mass spectrometry

## Abstract

Lipidomics aim to quantify lipid species in all kinds of samples, including tissues. To subject a fixed amount of sample to various workflows, tissue homogenates were frequently prepared at defined concentrations in water or by addition of organic solvents. Here, we investigated this first step of tissue lipidomics by quantitative flow injection analysis coupled to Fourier-Transform mass spectrometry (FTMS). The influence of sample concentration, solvent composition, and homogenization procedure on the recovery of lipids was studied in murine liver. Liver homogenates were prepared either by grinding tissue in liquid nitrogen or by bead-based homogenization. Ground samples were dissolved at different concentrations in water, methanol, and water/methanol = 1/1 (*v/v*). Here, lipid recovery depends on solvent composition and sample concentration. The recovery of nonpolar lipid classes, including triglycerides and cholesteryl ester, was decreased in methanolic homogenates. In contrast, due to superior dispersion of precipitates, bead-based homogenization resulted in efficient lipid recovery independent of the solvent composition. However, lipid distribution within samples, i.e., lipid content of supernatant and pellet following centrifugation, was altered substantially by solvent composition. In conclusion, accurate lipid quantification of tissue homogenates requires evaluation of solvent composition, sample concentration, as well as the homogenization method to guarantee efficient lipid recovery. Due to a potential loss of lipids, removal of precipitates by centrifugation prior to lipid extraction should be avoided.

## 1. Introduction

The field of lipidomics is a subset of metabolomics; it has emerged, along with technical advances, in mass spectrometry [1,2,3]. The typical workflow of lipidomics analysis comprises sample preparation, acquisition, processing, and interpretation of data [4,5]. Each of these steps needs an appropriate method that should be carefully evaluated to achieve accurate quantification of lipids [6]. To guide this development, Lipidomics Standard Initiative (LSI; https://lipidomics-standards-initiative.org/, accessed on 7 June 2021) was founded recently as a community-based effort [7]. Quantitative lipid species data provide the key to enhance the understanding of their biological functions and to investigate their changes in pathophysiology [8].

In this context, it is also important to preserve lipid composition during sample collection and processing. Lipid degradation can be induced by a variety of factors, such as chemical changes (e.g., oxidation) or ongoing metabolism (e.g., lipase activity). A review summarizing efforts to improve lipid stability was published recently [9]. Furthermore, the type of biological samples subjected to lipidomic analysis can be highly diverse and includes, among others, biofluids and solid samples, e.g., tissue. While the analysis of fluidic samples is generally straightforward, solid samples afford homogenization prior to lipidomic analysis in order to provide sufficient lipid extraction. 

Several techniques have been applied to physically disrupt tissue material, such as grinding frozen tissue with mortar and pestle [10], or more technically advanced processes, such as bead-based homogenization, e.g., the Precellys tissue homogenizer [11,12]. Grinding in liquid nitrogen has long been considered a gold standard for tissue homogenization, e.g., for isolation of high-quality mRNA, because sample heating is minimized during powdering. Furthermore, tissue grinding provides a homogenous powder representing the overall lipid composition, which is highly suitable for experiments or comparisons requiring identical source material. Nonetheless, due to its simplicity and throughput capabilities, bead-based homogenization methods are advantageous and in particular applied in studies with high sample numbers. Bead-based approaches are commonly directly performed in organic solvents optimized for a single workflow.

Frequently, homogenization of tissues is applied to generate a fluidic sample at a defined concentration [13], which provides several advantages in comparison to direct addition to the extraction [14,15]. Such samples permit a straightforward sample handling and allow the introduction into several workflows with a fixed amount of sample [16]. Furthermore, sampling of non-representative sample fractions can be avoided by subjecting larger sample portions to homogenization. For example, a zonal distribution of lipid species was described for liver tissue [17].

Several studies have evaluated the influence of tissue handling including homogenization on metabolite analysis. These studies typically focused on the total number of analytes/features detected or on a comparison of their signal intensities, but not on the concentration of lipids (reviewed in [10]). In the present study, we prepared homogenates from murine liver as fluidic samples, facilitating introduction into multiple workflows. Quantitative lipidomic analysis was applied to evaluate the effect of sample concentration, solvent composition, and homogenization procedure on lipid recovery and composition.

## 2. Results

### 2.1. Lipid Recovery and Composition Is Influenced by Concentration and Solvent Composition of Ground Liver Homogenates

**Figure 1:**
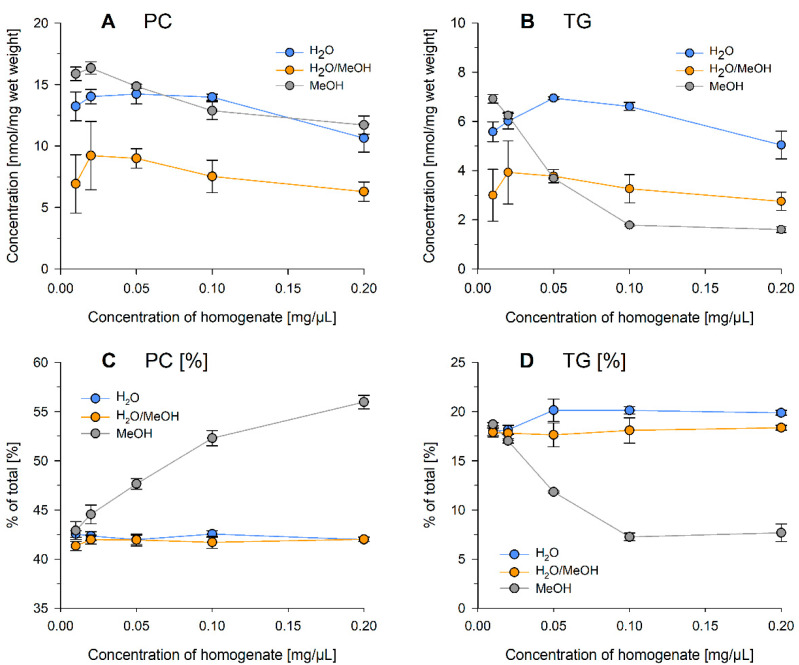
Concentration and lipid class fraction of PC (**A**,**C**) and TG (**B**,**D**) of mouse liver tissue homogenates suspended at concentrations of 0.01, 0.02, 0.05, 0.1, or 0.2 mg/µL in H_2_O, H_2_O/MeOH = 1/1 (*v/v*), or MeOH. The tissue was ground in liquid nitrogen with mortar and pestle. Displayed are mean +/− SD of a triplicate analysis.

Homogenization of tissues is commonly performed in water (or aqueous buffers), methanol, or methanol-containing solvents. Following this, we investigated, in the first step, the influence of solvent composition and concentration of the sample homogenate. Therefore, mouse liver tissue was ground with mortar and pestle in liquid nitrogen to provide a sufficient quantity of homogeneous source material. The powdered liver was suspended in H_2_O, H_2_O/MeOH = 1/1 (*v/v*), or MeOH at different concentrations to obtain fluidic samples. For all samples investigated in this study, a volume, representing 2 mg wet weight, was subjected to lipid extraction. Extraction was performed in the presence of internal standards (Table 1) using the protocol described by Bligh/Dyer [18]. Mass spectrometry analysis was performed by flow injection analysis coupled to Fourier-Transform mass spectrometry (FIA–FTMS) [19] and the following lipid classes were determined: cholesteryl ester (CE), diglycerides (DG), free cholesterol (FC), lysophosphatidylcholine (LPC), lysophosphatidylethanolamine (LPE), phosphatidylcholine (PC), phosphatidylethanolamine (PE), sphingomyelin (SM), and triglycerides (TG). To simplify matters, only PC and TG, as the most abundant polar and nonpolar lipid classes, are displayed in Figure 1. Overall, lipid recovery depended on sample concentration and decreased at higher concentrations. H_2_O/MeOH revealed the lowest recovery for all analyzed lipid classes (except TG in MeOH at high concentrations) with approximately 30–60% less compared to pure H_2_O (Figure 1A,B). While PC recovery followed similar trends in all solvents (Figure 1A), TG substantially decreased at higher concentrations in MeOH (Figure 1B). This led to a substantial concentration-depended shift in the lipid profiles of methanolic samples, including an increase of the PC fraction (Figure 1C). The lipid profiles in H_2_O and H_2_O/MeOH homogenates were similar and stable in the tested concentration range (Figure 1C,D; see Supplementary Figure S1 for all analyzed lipid classes). The species composition was similar for all three solvents and not affected by the concentration of the homogenate ( Supplementary Figures S2 and S3 for PC and TG species profile, respectively). 

### 2.2. Sample Inhomogeneity of Ground Liver Homogenates Influences Lipid Recovery

**Figure 2:**
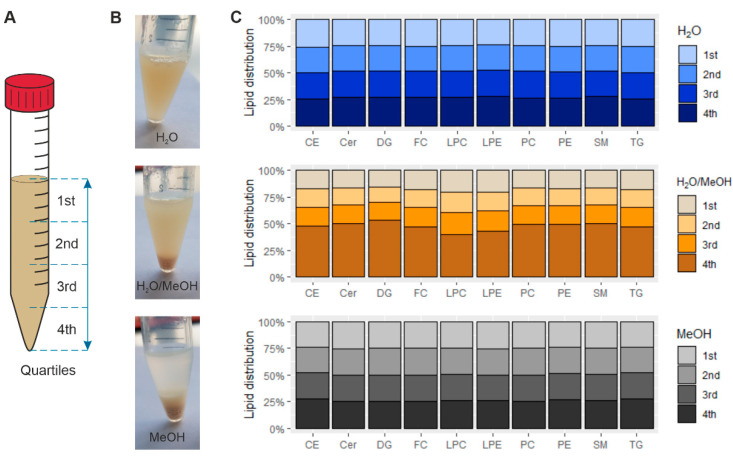
Evaluation of sample homogeneity of mouse liver tissue ground in liquid nitrogen and suspended in H_2_O, H_2_O/MeOH = 1/1 (*v/v*), or MeOH. (**A**) Fractionation of the quartiles by volume. (**B**) Mouse liver homogenates after 5 min sedimentation time. (**C**) Lipid distribution in the respective quartiles. The concentration of the homogenates was 0.05 mg/µL. Displayed is the mean of a triplicate analysis.

**Figure 3:**
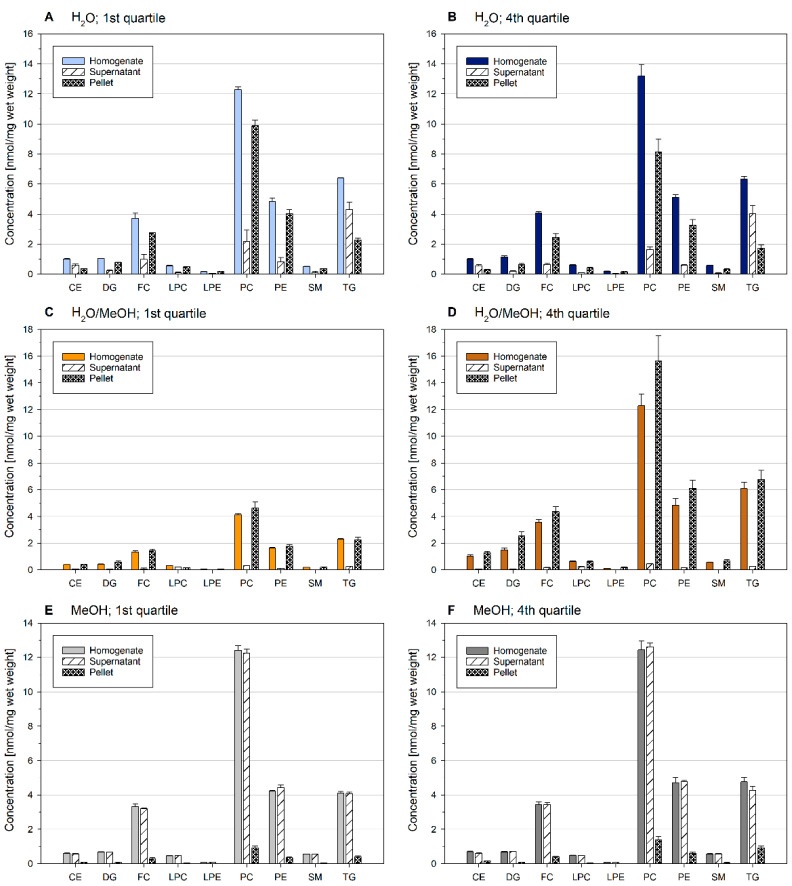
Lipid content of homogenate (prior centrifugation), supernatant, and pellet of the first and fourth quartile of mouse liver samples. Lipid distribution between quartiles is shown in Figure 2. The mouse liver was ground in liquid nitrogen and suspended in H_2_O (**A**,**B**), H_2_O/MeOH (**C**,**D**), or MeOH (**E**,**F**). The concentration of the homogenates was 0.05 mg/µL. Displayed are mean +/− SD (*n* = 3).

To further investigate solvent effects on liver homogenates (all dissolved at 0.05 mg/µL), samples were incubated on ice for 5 min to allow sedimentation. Subsequently, homogenates were separated into quartiles as shown in Figure 2A (for further details, see Section 4.4 Experimental Design). In contrast to aqueous samples, substantial protein precipitation was observed in MeOH containing solvents (Figure 2B). Lipid class concentrations for H_2_O and MeOH were ~25%/quartile indicating a homogenous distribution (Figure 2C). In contrast, solvation in H_2_O/MeOH resulted in enrichment in the bottom fraction. Of note, the lipid composition was identical for all quartiles of the same solvent. A decreased fraction of neutral lipids was again observed in MeOH (see also Figure 3). 

### 2.3. Lipid Content of Precipitates of Ground Liver Homogenates Depend on the Homogenization Solvent

Due to substantial protein precipitation upon MeOH addition, we asked whether these precipitates contain lipids. Therefore, the first and fourth quartiles were subjected to centrifugation. Lipid concentrations of the liver homogenate, as well as the supernatant and pellet, are shown in Figure 3. 

While pellets of H_2_O and H_2_O/MeOH contained a substantial amount of lipids, homogenates dissolved in MeOH (E and F) revealed only a minor lipid fraction. Here, almost all lipids remained in the supernatant. In aqueous samples, lipid distribution was related to lipid class polarity—polar lipid classes were mainly detected in the pellet fraction, whereas nonpolar TG and CE were mainly found within the supernatant. Of note, the summed concentration of supernatant and pellet matched very well the concentration of the aqueous homogenate. Samples dissolved in H_2_O/MeOH (C and D) contained almost no lipids in the supernatant. Although the concentration of lipids deviated substantially between the first and fourth quartiles in H_2_O/MeOH, lipid class profiles of the supernatant, and pellet were similar as for the other analyzed solvents and quartiles (data not shown). 

Taken together, these data demonstrate that lipid distribution within ground tissue samples is considerably affected by solvent composition. Furthermore, separation of precipitates prior to lipid extraction should be avoided.

### 2.4. Residual Lipid Content of Ground Liver Homogenates Depends on the Homogenization Solvent

**Figure 4:**
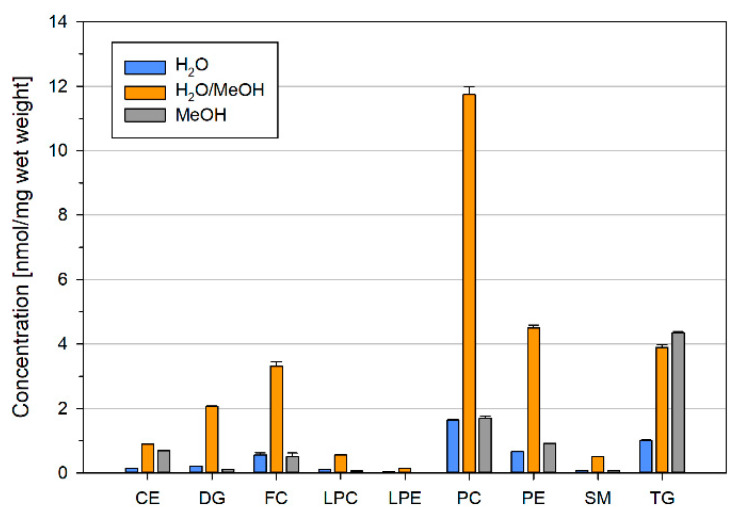
Lipid content of residual material. The mouse liver was ground in liquid nitrogen and suspended in H_2_O, H_2_O/MeOH, or MeOH. The sample homogenates were removed; tubes including residual precipitates were washed with a 2:1 mixture of MeOH/CHCl_3_ (*v/v*) and subjected to Bligh and Dyer extraction. Displayed are mean +/− SD (*n* = 3).

Another question was whether lipids could be lost due to adherence to the surfaces of tubes. Therefore, we added extraction solvent to empty sample tubes containing potential precipitates attached to the surfaces (Figure 4). Highest concentrations were recovered for H_2_O/MeOH, which matches well to the rather low total lipid content detected within the quartiles and the observed sedimentation tendency (Figure 2). The residual amount of lipids in H_2_O and MeOH was comparably low. Notably, we found an enrichment of neutral lipids (CE and TG) in the residues of MeOH, potentially explaining the observed altered lipid composition compared to H_2_O containing solvents.

### 2.5. Bead-Based Homogenization Improves Lipid Recovery Due to Enhanced Sample Dispersion

Next, we asked whether MeOH-induced precipitation might form aggregates, which are not accessible to organic solvents and, therefore, may cause a decreased lipid recovery. Thus, we examined whether mechanically bead-based homogenization of mouse liver tissue influences the degree of dispersion and lipid recovery. Moreover, we evaluated the effect of a detergent on lipid recovery by additionally investigating MeOH/H_2_O supplemented with 1% SDS. 

**Figure 5:**
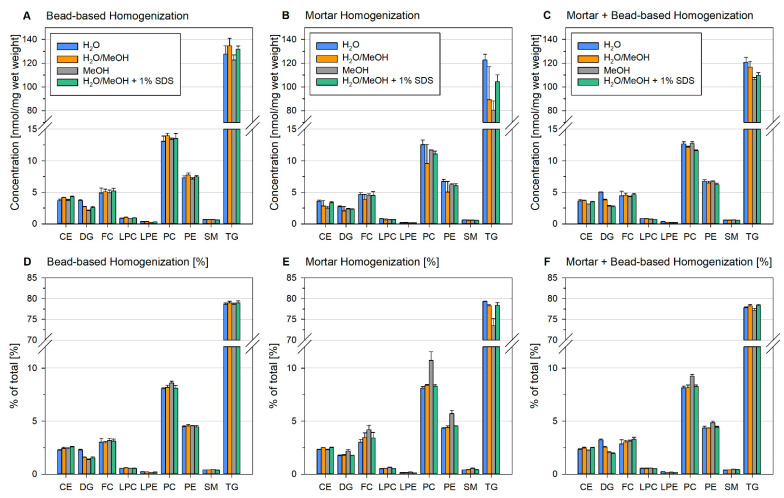
Influence of different homogenization procedures on quantification and lipid composition in mouse liver. Liver was subjected to bead-based homogenization (**A**,**D**), mortar homogenization (**B**,**E**), and mortar grinding followed by bead-based homogenization (**C**,**F**). The tissue (corresponding to 20–70 mg wet weight) was homogenized at a concentration of 0.05 mg/µL in a 2 mL cup in either H_2_O, H_2_O/MeOH = 1/1, H_2_O/MeOH = 1/1 +1% SDS, or MeOH. Displayed are mean +/− SD (*n* = 3).

Intriguingly, lipid recovery applying ceramic bead-based homogenization was similar for all solvents (Figure 5A,D). Solvation of ground liver (mortar homogenization) showed similar results as described before, with a reduced lipid recovery in MeOH/H_2_O and a reduced amount of nonpolar lipids in MeOH (Figure 5B,E). The addition of SDS resulted in a slightly increased recovery compared to pure MeOH/H_2_O. To investigate whether the diminished recovery of ground samples could be improved by enhanced dispersion, these samples were additionally subjected to bead-based homogenization (Figure 5C,F). This extra homogenization step improved lipid recovery significantly and only minor differences were observed between solvents. 

In summary, these data indicate that only a high degree of dispersion guarantees sufficient lipid recovery. Moreover, insufficient recovery can only affect some lipid classes, e.g., non-polar TG, resulting in an altered lipid composition (Figure 5D–F). 

### 2.6. Homogenization Solvent Influences Lipid Content of Sample Precipitates

**Figure 6:**
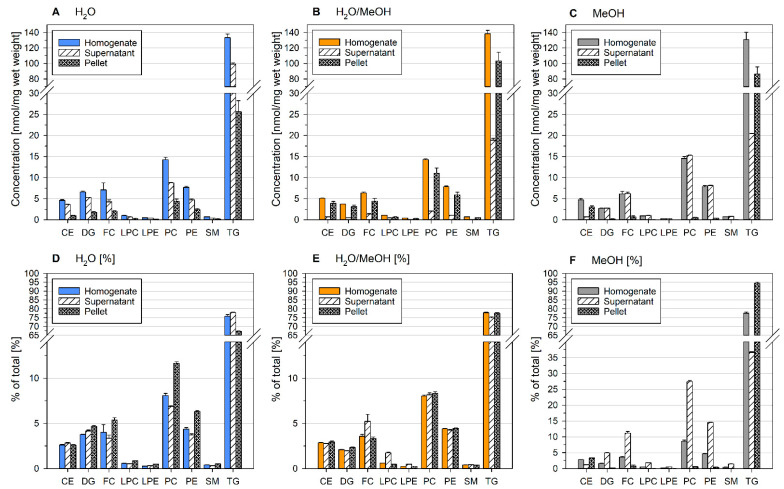
Lipid content of homogenate (prior centrifugation), supernatant, and pellet of mouse liver suspended after bead-based homogenization in H_2_O (**A**,**D**), H_2_O/MeOH (**B**,**E**), or MeOH (**C**,**F**). The tissue was homogenized at a concentration of 0.05 mg/µL in a 2 mL Precellys cup. Displayed are mean +/− SD (*n* = 3).

Finally, we checked whether solvent-dependent lipid distribution within samples differed between bead-based homogenization and ground samples (see Section 2.3. Lipid Content of Precipitates of Ground Liver Homogenates depend on the Homogenization Solvent). Therefore, bead-based homogenates, as well as their supernatants and pellets (after centrifugation), were subjected to lipid extraction and analysis (Figure 6). Of note, the liver sample used for bead-based homogenization had an almost 10-fold higher TG content compared to the liver used for grinding. However, the trends in the distribution of lipids matched very well the data presented in Figure 3. The lipid content within the pellet was highest for H_2_O/MeOH (Figure 6B) and lowest for MeOH (with exception for TG and CE; Figure 6C). However, the neutral lipid-rich liver sample disrupted by the bead-based homogenization revealed high concentrations of the nonpolar lipid classes CE and TG in MeOH precipitates (Figure 6C). This lipid class selectivity resulted in marked shifts of lipid profiles when sample supernatants were analyzed upon centrifugation (Figure 6F). Overall, these data demonstrate that removal of precipitates prior to lipid extraction could lead to deviations in lipid concentrations and profiles. Lipid content of precipitates may not only be related to sample concentration and homogenization solvent, but to the lipid content of the samples.

## 3. Discussion

In the present study, we investigated how the preparation of fluidic liver homogenates affects lipid recovery. We provide evidence that solvent composition, sample concentration, and homogenization strategy need to be evaluated carefully to guarantee sufficient lipid recovery and, consequently, accurate lipid concentrations and profiles. 

An issue is the formation of aggregates upon addition of organic solvents. The degree of aggregation is related to the concentration of the homogenate, which may explain an increased loss of neutral lipids at higher sample concentrations. This can lead to a substantial amount of lipids becoming inaccessible to the extraction solvent. On the other hand, if the concentration is too low, the adherence of lipids to the surface of tubes increases percentually, which could explain the tendency to lower lipid recovery (Figure 1). Such a loss of lipids could be more pronounced for precipitates with a high lipid content, as observed in H_2_O/MeOH, due to attachment of precipitates or direct adherence of lipids to surfaces. Thus, bead-based homogenization is advantageous compared to grinding and subsequent solvation of tissues because sample aggregation is minimized by bead agitation. This finding is also in agreement with a previous study on validation of lipid extraction using BUME lipid extraction of bead-based tissue homogenates [20]. Furthermore, the rapid motion of beads may mobilize residual material attached to the tube surfaces, which may benefit greatly by homogenizing directly in solvents. Taken together, we could show that appropriate sample dispersion is essential for sufficient recovery during lipid extraction.

Furthermore, the selection of the homogenization solvent could influence lipid recovery due to formation of lipid-containing precipitates. Precipitation of lipids clearly relates to their polarity. For example, homogenization in MeOH at high sample concentrations (Figure 1) or TG high content (Figure 6) results in reduced recovery and precipitation of nonpolar CE and TG, respectively. Consequently, lipid profiles may be shifted substantially due to insufficient lipid recovery or centrifugation steps. In conclusion, high sample concentration and centrifugation steps prior to lipid extraction should be avoided.

Another important aspect is sample stability, which was not considered in the present study. However, the addition of organic solvents could be advantageous for quenching of enzymatic activity and subsequent inhibition of lipid degradation [9]. In an accompanying study, the ratios of lipid classes, such as Cer/SM or LPE/PE, reflecting lipolytic activities, were used to investigate sample stability in various murine tissues [21]. Substantial lipolysis was observed in liver homogenates homogenized in H_2_O/MeOH = 1/1. However, the addition of SDS could significantly reduce lipolytic degradation in liver tissue, which represents, besides the increased lipid recovery in ground samples, an additional benefit for SDS addition. 

Sample stability and sufficient lipid recovery may be provided also by direct extraction of powdered tissue [14] or the addition of extraction solvent to bead-based homogenization [22]. However, such procedures could be expensive because internal standards need to be added for the entire tissue samples. Moreover, subjection of a defined sample amount to extraction and subsequent mass spectrometry analysis is more laborious for direct extraction compared to fluidic homogenates because it may need several cutting and weighing steps to adjust the amount of tissue. This comprises the additional risk of tissue warming and sample degradation. Furthermore, the addition of nonpolar organic solvents, such as chloroform to bead-based homogenization, may result in an increased chemical background leaching from plastics. Generation of fluidic homogenates at a defined concentration further has the advantage that those samples could be introduced easily to various workflows. Moreover, such kind of samples provide a uniform chemical background due to a fixed sample volume facilitating simple background corrections by analysis of blank samples. Homogenization of larger sample portions could also avoid misinterpretation due to sampling of zonal or non-representative sample parts [17]. 

Given these points, we apply bead-based homogenization to generate fluidic liver homogenates. Our results demonstrate that lipid recovery and preanalytical stability [21] is sufficient in H_2_O/MeOH = 1/1 supplemented with 1% SDS at a concentration of 0.05 mg wet weight/µL. However, we would like to emphasize that these conditions should not simply be transferred to other tissues without proper evaluation of preanalytics. For example, SDS addition led to LPE generation in murine lung and spleen samples [21]. Respective evaluations should include both recovery and stability of lipids, which could be provided also by other stabilization methods, such as heat inactivation [23]. 

In summary, we could provide evidence that sample concentration, composition of the solvent, as well as homogenization efficiency, i.e., degree of dispersion, are crucial for sufficient lipid recovery from tissue homogenates. Importantly, sample pellets should only be separated prior to lipid extraction when lipid loss could be excluded. Beside efficient extraction, application of an appropriate method for lipid species quantification and consideration of preanalytical stability are required to achieve accurate lipid concentrations [5,9]. 

## 4. Materials and Methods

### 4.1. Chemicals and Lipid Standards

Chloroform and 2-propanol were purchased from Roth (Karlsruhe, Germany) and methanol from Merck (Darmstadt, Germany). All solvents were HPLC grade. Nuclease-free water was obtained from B. Braun (Melsungen, Germany). Ammonium formate, sodium dodecyl sulfate (SDS), and cholesteryl ester (CE) standards were purchased from Sigma-Aldrich (Taufkirchen, Germany). Moreover, [25,26,26,26,27,27,27-D_7_]-cholesterol was acquired from Cambridge Isotope Laboratories (Andover, MA, USA) with isotope purity higher than 98%. Triglyceride (TG) and diglyceride (DG) standards were purchased from Larodan (Solna, Sweden). Phosphatidylcholine (PC), ceramide (Cer), sphingomyelin (SM), lysophosphatidylcholine (LPC), and lysophosphatidylethanolamine (LPE) standards were purchased from Avanti Polar Lipids (Alabaster, AL, USA). The composition of the added internal standard mixture is depicted in Table 1.

### 4.2. Biological Samples

Liver tissue was obtained from mice of strain C57BL/6J with a low-density lipoprotein receptor (LDLR) knockout. Animals used in this study were residuals within crossbreeding. The tissue was perfused with PBS and snap frozen in liquid nitrogen. Procedures were approved by the University of Regensburg Laboratory Animal Committee and complied with the German law on animal protection and the Institute for Laboratory Animal Research Guide for the Care and Use of Laboratory Animals. Experiments were conducted according to institutional and governmental regulations for animal use.

### 4.3. Preparation of Tissue Homogenates

#### 4.3.1. Mortar Homogenization

The frozen liver was cut in smaller pieces with a sharp scalpel. Afterwards, the tissue pieces (about 1 g wet weight in total) were transferred to a stainless steel mortar and immediately doused with liquid nitrogen. The frozen pieces were ground with a pestle upon reaching a homogenous powder-like state (~5 min). During this procedure, liquid nitrogen was continuously added to avoid tissue thawing. The powder was aliquoted and weighed to determine the wet weight. Different solvents H_2_O, H_2_O/MeOH = 1/1 (*v/v*), H_2_O/MeOH = 1/1 +1% SDS, or MeOH were added to suspend at the respective concentration. The homogenate was vortexed at 3200 rpm for 10 s and another 10 s prior to sample taking.

#### 4.3.2. Bead-Based Homogenization

A small piece, of approximately 20–70 mg, was cut off a frozen liver and transferred in a Precellys cup with ceramic beads (V = 2 mL). Different solvents H_2_O, H_2_O/MeOH = 1/1 (*v/v*), H_2_O/MeOH = 1/1 +1% SDS, or MeOH were added to suspend the samples at the concentration of 0.05 mg/µL. The sample was directly homogenized in the previously added solvent with a Precellys^®^ 24 tissue homogenizer from Bertin Instruments (Berlin, Germany). The homogenizer was operated at 5000 rpm, two cycles of 15 s run time, and a 60 s break interval between both cycles.

### 4.4. Experimental Design

**Figure 7:**
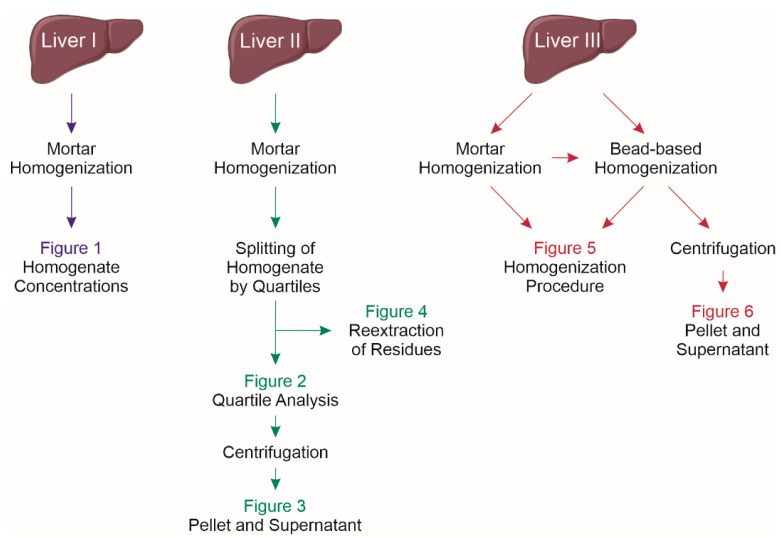
Overview of the experimental workflow.

Three murine liver samples were used to investigate the homogenization procedure, solvent effects, and effect of centrifugation (Figure 7). For quartile analysis (liver II), the homogenate was kept on ice for 5 min. Afterwards, the total volume was divided by four and each quartile was carefully transferred into a new tube. The residual material in the initial tubes was recovered with MeOH/CHCl_3_ (2:1, *v/v*) and submitted to lipid extraction (Figure 4). The quartile tubes were vortexed before sample taking (Figure 2). Next, these tubes were centrifuged for 10 min at 4000 rpm (17,860× *g*) to separate the supernatant and pellet (Figure 3). The supernatant was transferred to a new tube before sample taking. The pellet was resuspended in MeOH/CHCl_3_ (2:1 *v/v*) and subjected to lipid extraction as described in Section 4.5 Lipid Extraction. 

The comparison of homogenization procedures was performed with liver III. A small piece was cut off a frozen liver and submitted to bead-based homogenization (described in Section 4.3.2 Bead-Based Homogenization) before the residual tissue was ground with mortar and pestle (described in Section 4.3.1 Mortar Homogenization). The respective homogenates were submitted to lipid extraction (Figure 5). Subsequently, the bead-based homogenate was transferred to a new Precellys cup and centrifuged for 10 min at 13,000 rpm (16,000× *g*) to separate supernatant and pellet (Figure 6). The supernatant was transferred to a new tube before submission to lipid extraction. To the pellet, MeOH/CHCl_3_ (2:1 *v/v*) as well as ceramic beads were added. The pellet was homogenized at 5000 rpm, two cycles of 15 s run time, and a 60 s break interval between both cycles before sample taking.

### 4.5. Lipid Extraction

Lipid extraction was performed in 15 mL glass centrifuge tubes. First, 50 µL of a prepared internal standard stock solution (Table 1) was added to the tubes. The solvent was removed by vacuum centrifugation. Afterwards, an amount of 2 mg liver tissue (wet weight) homogenized in either H_2_O, H_2_O/MeOH = 1/1 (*v/v*), H_2_O/MeOH = 1/1 +1% SDS, or MeOH was added. Sample material from pellet resuspension (Figure 3 and Figure 6) and recovery of residues (Figure 4) was suspended in MeOH/CHCl_3_ (2:1 *v/v*). The concentration of the liver homogenates was adjusted to 0.05 mg/µL resulting in a sample volume of 40 µL. Data presented in Figure 1 were generated from liver homogenates adjusted to 0.2, 0.1, 0.05, 0.02, and 0.01 mg/µL and the corresponding sample volume used for extraction was 10, 20, 40, 100, and 200 µL, respectively. Of note, for volume spikes >40 µL, the solvent composition of the extraction (H_2_O, MeOH, and CHCl_3_) was reduced accordingly. 

The lipid extraction was performed according to the protocol described by Bligh and Dyer [18]. A volume of 0.8 mL H_2_O and 3 mL MeOH/CHCl_3_ (2:1 *v/v*) was added to each sample. The suspension was mixed by vortexing and incubated for 1 h at room temperature. Subsequently, 1 mL H_2_O and 1 mL CHCl_3_ was added to obtain a final solvent ratio of 1.8:2:2 for H_2_O:MeOH:CHCl_3_. Samples were mixed and centrifuged for 10 min at 4000 rpm (17,860× g) inducing phase separation. A volume of 500 μL of the chloroform phase was transferred into a sample vial by a pipetting robot (Tecan Genesis RSP 150, Männedorf, Switzerland) and vacuum dried. The residues were dissolved in 1 mL of 7.5 mM ammonium formate in chloroform/methanol/2-propanol (1:2:4 *v/v/v*).

### 4.6. Direct Flow Injection High Resolution MS

Lipid quantification was performed by direct flow injection on a Q Exactive Hybrid Quadrupole-Orbitrap Mass Spectrometer (Thermo Fisher Scientific, Bremen, Germany) equipped with a heated electrospray ionization (ESI) source. A detailed description of the method was published recently [19]. Positive ion mode FTMS data (TG, DG, and CE as ammoniated adducts) were recorded in the *m/z* range of 500–1000. CE species were corrected for their response [24]. MS/MS was applied for the determination of free cholesterol (FC) by multiplexing (MSX) [M+NH_4_]^+^ ions of cholesterol and D_7_-cholesterol [24]. Negative ion mode FTMS data were recorded in the *m/z* range of 400–650 for LPE as [M-H]^−^ and LPC as [M+HCOO]^−^, and *m/z* in the range of 520–960 for Cer, SM, and PC quantification as [M+HCOO]^−^. All data were acquired in profile mode with a target resolution of 140,000 (at *m/z* 200). Lipid species annotation is based on the latest update of the shorthand notation [25].

## Figures and Tables

**Table 1:** Composition of the internal standard mixture. A volume of 50 µL was added per sample.

Species	Molecular Weight	Stock Solution	Spiked Amount	
	(g/mol)	(µg/mL)	(ng/sample)	(nmol/sample)
CE 17:0	638.60	10	500	0.78
CE 22:0	708.68	10	500	0.71
Cer 32:1;O2	509.48	1	50	0.098
Cer 35:1;O2	551.53	1	50	0.091
DG 28:0	512.44	5	250	0.49
DG 40:0	680.63	5	250	0.37
[D7]FC	393.40	75	3750	9.5
LPC 13:0	453.29	1	50	0.11
LPC 19:0	537.38	1	50	0.093
LPE 13:0	411.24	1	50	0.12
PC 28:0	677.50	25	1250	1.8
PC 44:0	901.75	25	1250	1.4
PE 28:0	635.45	10	500	0.79
PE 40:0	803.64	10	500	0.62
SM 30:1;O2	646.50	10	500	0.77
TG 51:0	848.78	18	900	1.1
TG 57:0	932.88	18	900	0.96

## Data Availability

Data are contained within the article or supplementary material.

## Supplementary Materials

The following are available online at https://www.mdpi.com/article/10.3390/metabo11060365/s1, Figure S1: Lipid class composition of mouse liver tissue suspended at concentrations of 0.01, 0.02, 0.05, 0.1, or 0.2 mg/µL in (A) H_2_O, (B) H_2_O/MeOH = 1/1 (*v/v*), or (C) MeOH; Figure S2: Phosphatidylcholine (PC) species profile of mouse liver tissue suspended at concentrations of 0.01, 0.02, 0.05, 0.1, or 0.2 mg/µL in (A) H_2_O, (B) H_2_O/MeOH = 1/1 (*v/v*), or (C) MeOH; Figure S3: Triglyceride (TG) species profiles of mouse liver tissue suspended at concentrations of 0.01, 0.02, 0.05, 0.1, or 0.2 mg/µL in (A) H_2_O, (B) H_2_O/MeOH = 1/1 (*v/v*), or (C) MeOH.
