# Supplementary material for: Accurate Lipid Quantification of Tissue Homogenates Requires Suitable Sample Concentration, Solvent Composition, and Homogenization Procedure—A Case Study in Murine Liver
Source: Metabolites. 2021 Jun 8;11(6):365. doi: 10.3390/metabo11060365 (PMC8228350; doi:10.3390/metabo11060365)
Supplement: Supplementary file 1 [file metabolites-11-00365-s001.zip › metabolites-1238830-supplementary.pdf]

metabolites

MDPI

Supplemental Material

Accurate Lipid Quantification of Tissue Homogenates Requires Suitable Sample Concentration, Solvent Composition, and Homogenization Procedure – a Case Study in Murine Liver

Marcus Höring [1], Sabrina Krautbauer [1], Louisa Hiltl [1], Verena Babl [1], Alexander Sigruener [1], Ralph Burkhardt [1] and Gerhard Liebisch [1,*]

[1] University Hospital of Regensburg, Institute of Clinical Chemistry and Laboratory Medicine, Franz-Josef-Strauß-Allee 11, 93053 Regensburg, Germany;
marcus.hoering@ukr.de (M.H.); sabrina.krautbauer@ukr.de (S.K.); louisa.hiltl@stud.uni-regensburg.de (L.H.); verena.babl@klinik.uni-regensburg.de (V.B.); alexander.sigruener@ukr.de (A.S.); ralph.burkhardt@ukr.de (R.B.)
* Correspondence: gerhard.liebisch@ukr.de; Tel.: +49941/9446240

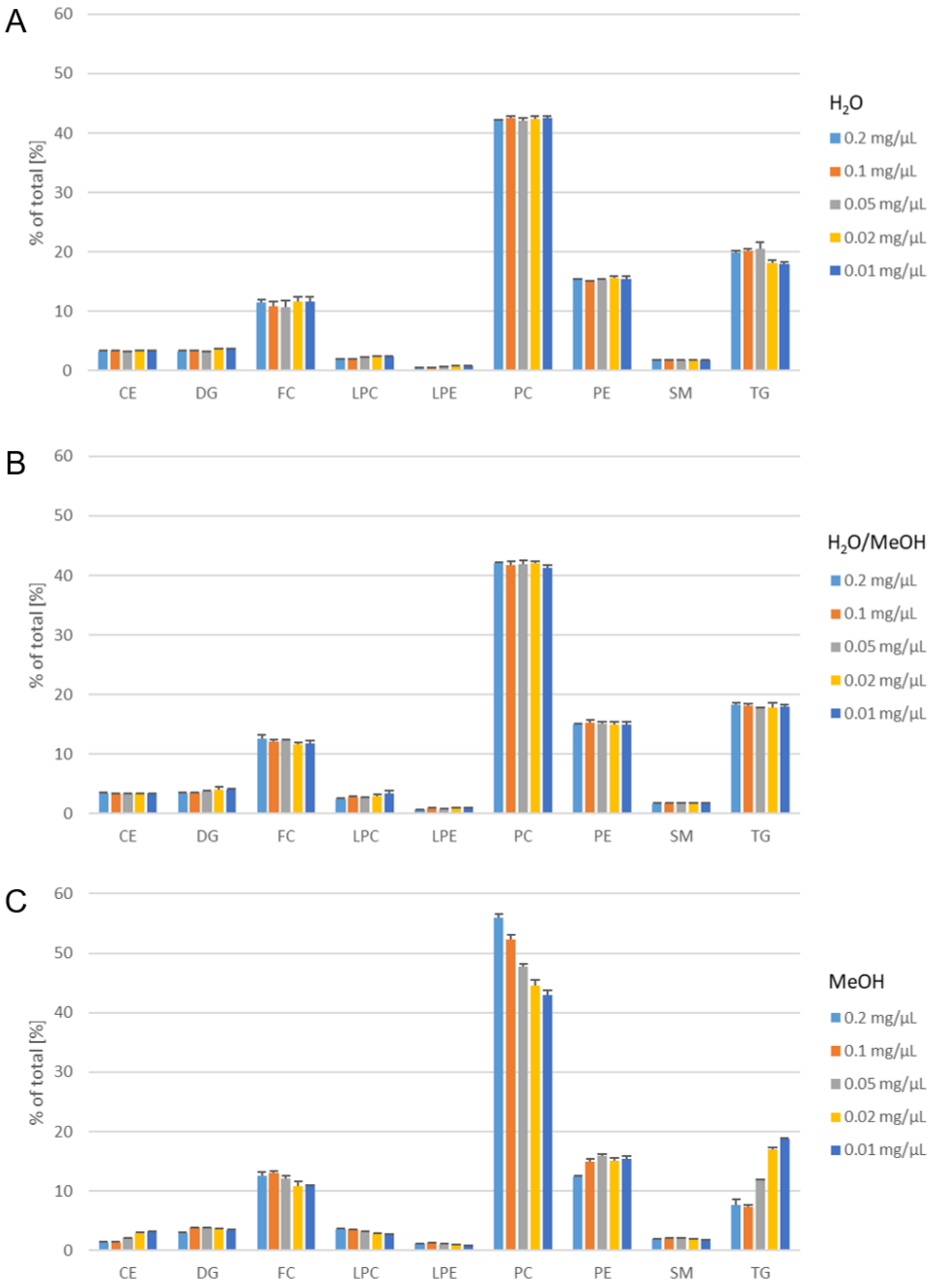

Figure S1. Lipid class composition of mouse liver tissue suspended at concentrations of 0.01, 0.02, 0.05, 0.1, or 0.2 mg/µL in (A) H_2O, (B) H_2O/MeOH = 1/1 (v/v), or (C) MeOH. The tissue was ground in liquid nitrogen with mortar and pestle. Displayed are mean +/- SD of a triplicate analysis.

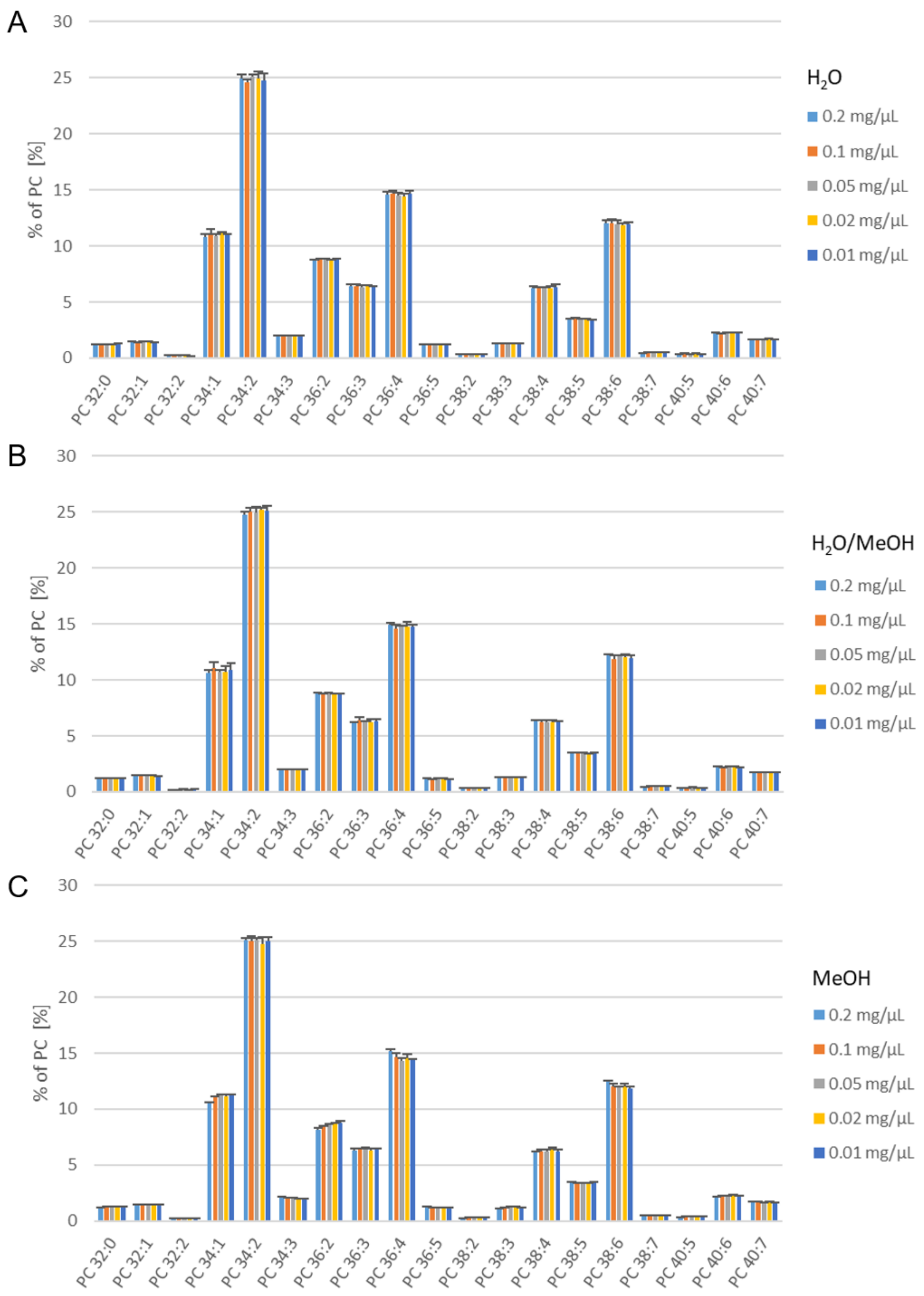

Figure S2. Phosphatidylcholine (PC) species profile of mouse liver tissue suspended at concentrations of 0.01, 0.02, 0.05, 0.1, or 0.2 mg/μL in (A) H_2O, (B) H_2O/MeOH = 1/1 (v/v), or (C) MeOH. The tissue was ground in liquid nitrogen with mortar and pestle. Displayed are mean +/- SD of a triplicate analysis.

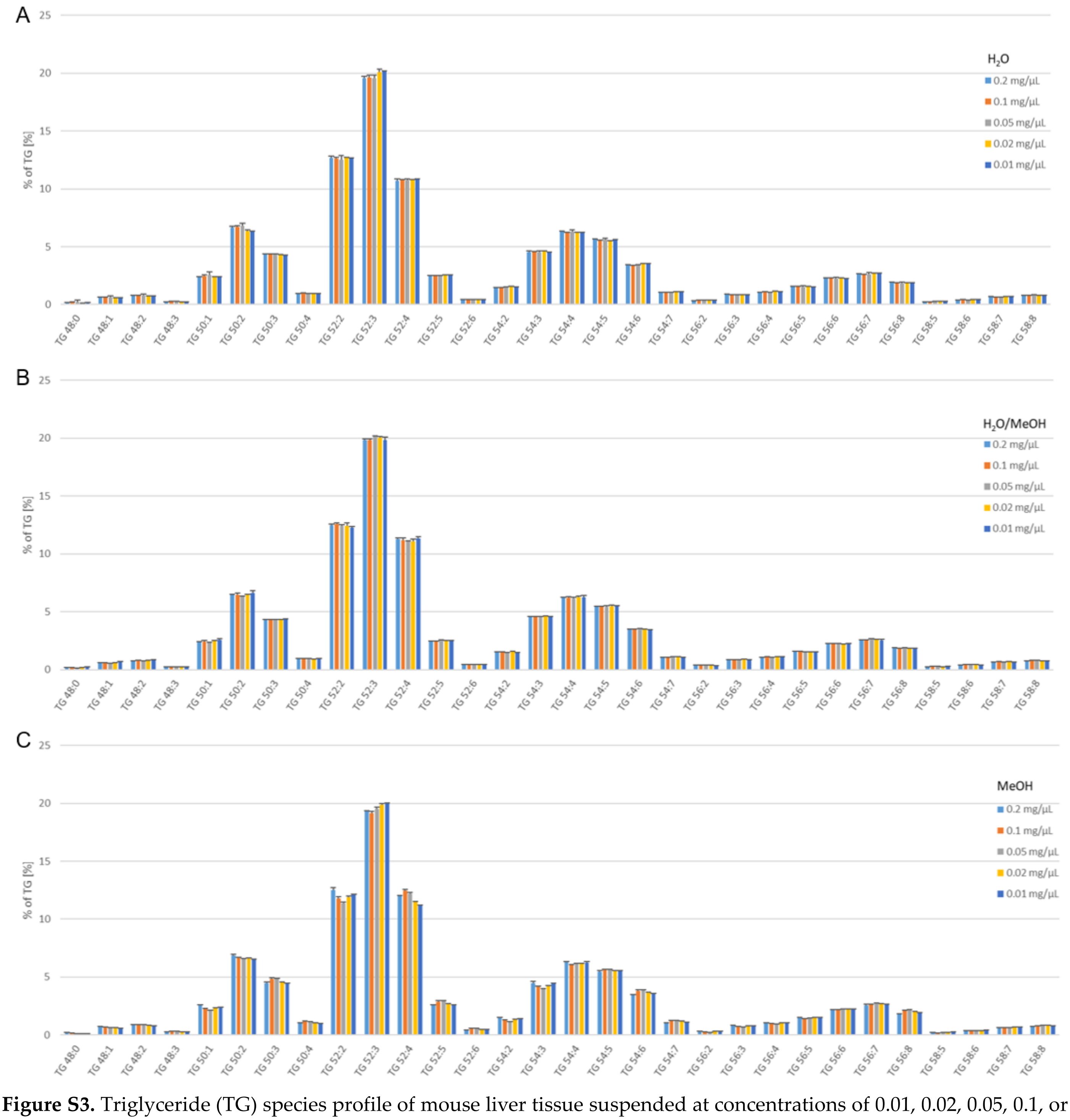

Figure S3. Triglyceride (TG) species profile of mouse liver tissue suspended at concentrations of 0.01, 0.02, 0.05, 0.1, or 0.2 mg/µL in (A) H_2O, (B) H_2O/MeOH = 1/1 (v/v), or (C) MeOH. The tissue was ground in liquid nitrogen with mortar and pestle. Displayed are mean +/- SD of a triplicate analysis.
